# A Chronic, Solitary Skin Ulcer Owing to Sporotrichosis

**DOI:** 10.4269/ajtmh.26-0146

**Published:** 2026-09-22

**Authors:** Nicolas Antunez de Mayolo, Ciro Maguiña-Vargas, Omayra Chincha

**Affiliations:** ^1^ Instituto de Medicina Tropical Alexander von Humboldt, Universidad Peruana Cayetano Heredia, Lima, Peru;; ^2^ Departamento de Enfermedades Infecciosas, Tropicales, y Dermatologicas, Hospital Nacional Cayetano Heredia, Lima, Peru

A 50-year-old woman from Ayacucho in the Peruvian highlands presented to the outpatient clinic with a 3-year history of a painless ulcerated lesion on her right knee. The patient denied prior trauma. The lesion initially appeared as a small papule, which slowly enlarged over time. On physical examination, a 5-centimeter diameter ulcer with crusting and suppuration was observed without regional adenopathy (Figure 1A).

**Figure 1.**
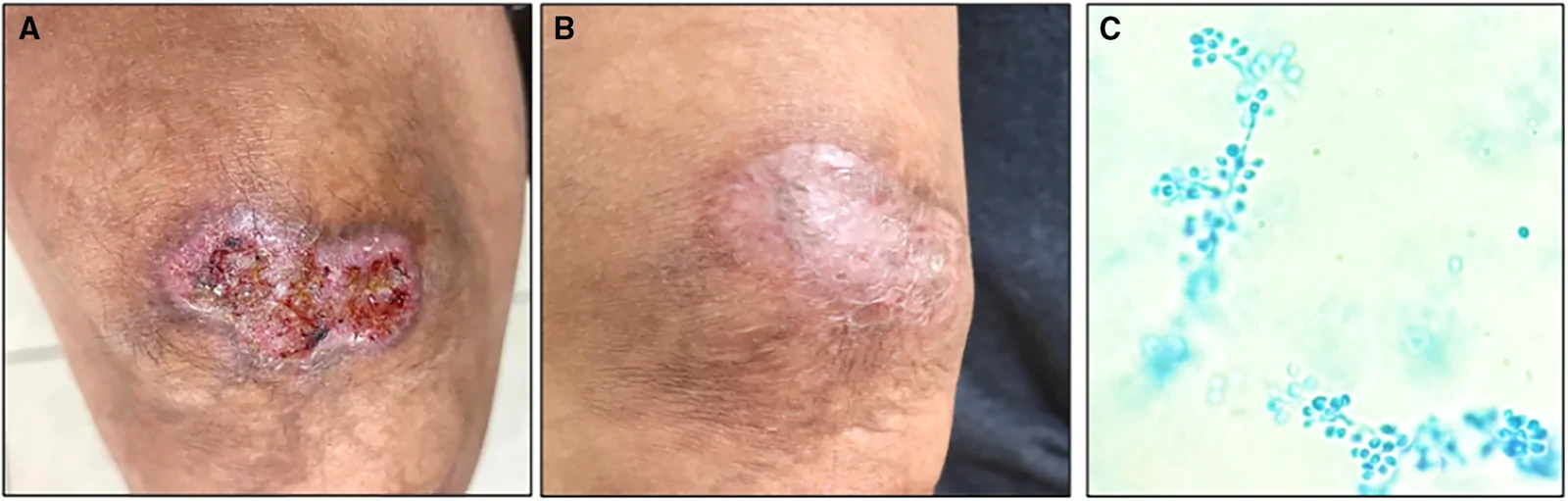
(**A**) Ulcerative lesion in the right knee. (**B**) Clinical improvement after 6 months of treatment. (**C**) Lactophenol cotton blue preparation showing the bouquet-like 54 arrangement of microconidia from the culture of lesion scrapings.

She was initially misdiagnosed with cutaneous leishmaniasis based on local epidemiology. Despite a negative direct Giemsa-stained smear, she received antimonial therapy on different occasions without clinical improvement.

Direct mycological examination using 10% potassium hydroxide was negative; fungal culture on peptone dextrose agar supplemented with chloramphenicol and cycloheximide was positive for *Sporothrix* spp. Colonies demonstrated the bouquet-like arrangement of microconidia (Figure 1C), and species identification as *S. schenckii* was confirmed by calmodulin gene sequencing. Treatment was started with itraconazole 200 mg daily for 6 months. Figure 1B shows the clinical improvement of the lesion after treatment, with a residual hypopigmented scar without ulceration.

Sporotrichosis is a fungal infection affecting humans and animals, and it is the most prevalent subcutaneous mycosis in Latin America.^1^ In Peru, the disease is endemic, particularly in the highlands and jungle, with an area of hyperendemicity located in Abancay.^2^ However, few studies have performed species-level identification, and all isolates reported to date have been identified as *S. schenkii*.^3^ In contrast to Brazil and other countries in the region, where *S. brasiliensis* has emerged as a major cause of cat-associated zoonotic transmission and has been linked to outbreaks,^4^ such transmission has not been reported in Peru. Other pathogenic species, including *S. globosa*, have also been reported in Latin America.^1^ Although the most common clinical manifestation of sporotrichosis is lymphocutaneous spread with the development of nodules along lymphatic tracts, the disease may also present as a solitary ulcerative lesion classified as the fixed cutaneous form.^5^ Sporotrichosis should be considered in the differential diagnosis of a chronic solitary ulcerative lesion, particularly in endemic areas.
